# Extract of *Euterpe oleracea* Martius Stone Presents Anticonvulsive Activity *via* the GABAA Receptor

**DOI:** 10.3389/fncel.2022.872743

**Published:** 2022-05-11

**Authors:** Nilton Akio Muto, Moisés Hamoy, Chryslen Brenda da Silva Ferreira, Akira Otake Hamoy, David Cristian Rodrigues Lucas, Vanessa Jóia de Mello, Hervé Rogez

**Affiliations:** ^1^Centre for Valorization of Amazonian Bioactive Compounds (CVACBA), Federal University of Pará (UFPA), Belém, Brazil; ^2^Laboratory of Pharmacology and Toxicology of Natural Products, Institute of Biological Sciences of Federal University of Pará (ICB-UFPA), Belém, Brazil

**Keywords:** seizure, anticonvulsant, EEOS, DZP, GABAA receptor

## Abstract

Epilepsy is one of the most common neurological diseases globally, resulting from a disorder in brain activity. This condition can be triggered by birth trauma, traumatic brain injury (TBI), infections of the brain and stroke. More than 70 million people suffer seizures caused by neurological abnormalities. Approximately 80% of all epileptic patients reside in low-income conditions or in developing countries, and over 75% of patients do not receive proper treatment. Our previous study found an anticonvulsant property of an extract of *Euterpe oleracea* stone (EEOS) that caused myorelaxation, sedation, and cardiac and respiratory depression after intraperitoneal administration. The present study investigated through electroencephalographic (EEG) profiling the anticonvulsant protective properties of EEOS in induced convulsing rats. Male Wistar rats were treated with EEOS (300 mg/kg), diazepam (DZP) (5 mg/kg), pentylenetetrazol (PTZ) (60 mg/kg) and flumazenil (FMZ) (0.1 mg/kg) by intraperitoneal (i.p.). Electrodes implanted on the dura mater provided EEG data in which EEOS suppressed seizure deflagration caused by PTZ. In addition, EEOS presented no significant difference in comparison to DZP, which has the same mechanism of action. After FMZ injection, a GABAA receptor antagonist blocked the anticonvulsive effect in both the DZP and EEOS groups, suggesting that EEOS exerts it action on the GABAA receptor at the benzodiazepine (BDZ) subunit.

## Introduction

Epilepsy is a neurological disease of the central nervous system (CNS). It is one of the most common neurologic diseases worldwide, and it has many causes, including head injuries (traumas, strokes), alcohol and drug abuse, tumors, and other neurological diseases. It is characterized by a neurological illness in the brain that spreads throughout the body in brief episodes of involuntary movements involving specific parts or the entire body. The irregular electrical activity can be accompanied by a loss of consciousness and a loss of control of intestinal or bladder function (World Health Organization, 2019). Seizure episodes are associated with a paroxysmal depolarizing shift with perturbation of the neuronal membrane voltage in a brain cell group (Kubista et al., 2019). Seizures can present in many ways, from the briefest loss of attention and muscle tremors to severe and extended seizures adversely impairing the quality of life of patients, affecting them both psychologically and socially. Their frequency varies according to the history of the disease and whether it is untreated or suboptimally treated epilepsy (Guekht et al., 2007).

Current anticonvulsant drugs for seizures are not fully effective, and approximately one out of three patients with epilepsy have refractory seizures (Arrifano et al., 2018). Many of those patients, approximately 80%, live in developing countries, and geographic isolation and low income and purchasing power are the main barriers to accessing proper treatment in these countries (Newton and Garcia, 2012). One study estimated that more than three-quarters of patients with severe epilepsy, after treatment for 6 years, no longer need to take antiepileptic medicines (gabapentin, lamotrigine, or vigabatrin), thus being free from seizures (Nsour et al., 2000). For these reasons, the development of novel AEDs (anti-epileptic drugs) from medicinal plants may represent an important resource for the treatment of epilepsy.

Açai (*Euterpe oleracea* Martius) is a typical palm found in the floodplains, land, and swampland soils of eastern Amazonian. It is labeled a “superfruit” because of its high levels of antioxidants, fat, fiber, and relatively low sugar. Its juice is consumed daily in northern Brazil as a staple food, and it has recently attracted attention in other regions (Bichara and Rogez, 2011).

Acai juice has been extensively studied because of its health benefits and bioactive compounds such as anthocyanins, fatty acids, and other trace minerals (which include copper, chromium, iron, magnesium, manganese, potassium, and phosphorus). Even though the juice is the hallmark part of the fruit, the stone represents more than 80% of the drupe weight, and little is known about its properties (Barros et al., 2015).

Although açai stones are not edible its high antioxidant capacity has been studied for new applications through the treatment of diabetes and lung cancer (De Bem et al., 2018; Martinez et al., 2018). Extracts of *Euterpe oleracea* stone (EEOS) analyzed through HPLC/MS exhibited high levels of polyphenols such as caffeic acid, procyanidin, catechin, polymeric proanthocyanidins, and cinnamtannin, followed by traces of other phenolic compounds (Muto et al., 2021).

Catechins, epicatechins, and polymeric proanthocyanidins in EEOS have been associated with vasodilating, antihypertensive, and antioxidant activities. Additionally, it has been shown that *Euterpe oleracea* pulp exhibits antiepileptic properties in mice and benefits the metabolism of patients with diabetes associated with hypertension (Souza-Monteiro et al., 2015; De Bem et al., 2018). Furthermore, our previous results demonstrated that EEOS has strong sedative and myorelaxant effects similar to benzodiazepine-based drugs (BDZs) (Muto et al., 2021).

Benzodiazepine drugs act on the CNS, modulated by γ-aminobutyric acid (GABA) receptors, which are the chief inhibitory neurotransmitters of the CNS with glutamate acting as a precursor (Da Settimo et al., 2007). Although there are many studies involving herbs or medicinal plants for the treatment of epilepsy, most of them are restricted to the use of crude extracts or isolated fractions (Nsour et al., 2000; Siqueira et al., 2008). Due to the complexity of these extracts, few studies have investigated the mechanisms associated with their antiepileptic actions, which play a significant role in the understanding and development of novel AEDs.

Thus, this article aimed to investigate and elucidate by electroencephalographic (EEG) profiling the anticonvulsant properties of extracts of açai stones [*Euterpe oleracea* stone (EEOS)] in animal models of the Wistar lineage.

## Materials and Methods

### From Drupe to the Extract of *Euterpe oleracea* Stone

We used the EEOS solution from açai stones in our study, as previously described (Muto et al., 2021). In short, after pulping açai fruits, 100 g of açai stones was washed and dried at 105°C for 24 h. Stones were ground (16 mesh), shaken with 200 mL of ethanol for 2 h, and macerated at 4°C for 10 days in dark bottles. After maceration, the extract was filtered and roto evaporated under low pressure at 40°C. The extraction yield was 300 mg of dried extract from 35 mL. The dried extract was stored at −20°C until use.

### Determination of the Phytochemistry Profile of Extracts of *Euterpe oleracea* Stone

The phytochemistry profile of EEOS was previously determined by the colorimetric method (total phenolic compounds, flavanols, flavonols, and proanthocyanidins) as well as by liquid chromatographic analysis (LC-ESI-IT-MS/MS) (Muto et al., 2021).

### Animals

Male Wistar rats aged between 10 and 12 weeks (160–200 g) were kept in a controlled environment (25 ± 2°C; 12 h light/dark cycle) with *ad libitum* access to food and water. In total, 81 animals were used, divided into nine groups (*n* = 9 for each group). The groups were the behavioral group, control group, diazepam (União Química, Embu-Guaçu, Brazil) (DZP)-treated group, EEOS-treated group, and the pentylenetetrazol (Sigma-Aldrich, St. Louis, United States) (PTZ)-treated group. To control seizures we used: DZP + PTZ, EEOS + PTZ, followed by seizure deflagration with flumazenil (FMZ) (Laboratorio Teuto, Goiânia, Brazil).

Animals were treated following protocols approved by the Committee for Ethics in Experimental Research with Animals of Federal University do Pará (Brazil; license n° 1089220518) (8th Edition; 2011).^1^

### Behavioral Characterization of Animals After Administration of Extracts of *Euterpe oleracea* Stone, Diazepam, and Pentylenetrazol

The behavioral group used three groups: positive control application of 0.9% saline solution at a dose of 0.1 ml/100 g 10 min before the administration of PTZ at a concentration of 100 mg/mL (60 mg/kg, i.p.) for standardization of the seizures. The group with DZP had it administered 5 mg/kg i.p. 10 min before the application of PTZ 60 mg/kg i.p., and the third group received EEOS at 300 mg/kg i.p. 10 min before the administration of PTZ at a dose of 60 mg/kg i.p. (Muto et al., 2021). Behavioral analyses of the seizures were performed with continuous monitoring.

The behavioral changes during the seizures have six stages: 0: No behavioral change after PTZ administration; I: The animal has immobility; II: The animal exhibits rapid vibratory movements and a feathered tail; III: The animal presents isolated clonic seizures of one or more limbs, especially the anterior limbs; IV: The animal has generalized clonic seizures, abundant salivation, and transient loss of posture reflex; V: The animal presents tonic-clonic seizures (Great-mal), loss of posture reflex and death from respiratory arrest (Souza-Monteiro et al., 2015).

The latencies and behaviors were observed 10 min after the administration of DZP and EEOS, caused by the administration of PTZ, during 30 min of observation.

### Electroencephalogram Analyses

#### Electrode Placement Surgery

Animals were anesthetized by intraperitoneal administration of ketamine hydrochloride (50 mg/kg) (Köing Laboratory, Santana de Parnaíba, SP, Brazil) and xylazine hydrochloride (5 mg/kg) (Vallée laboratory, Montes Claros, MG, Brazil). After interruption of the corneal reflex, the animals were immobilized in a stereotactic apparatus and the skull was exposed. Two bilateral holes were made in the skull with a dental drill. Stainless steel electrodes (1.0 mm diameter tip exposure) were placed on the dura mater above the frontal cortex at bregma coordinates −0.96 mm and ± 1.0 mm lateral (Paxinos and Watson, 2009). A small screw was placed in the occipital region, and the electrodes were affixed with dental acrylic cement.

#### Electroencephalographic Records

The animals were anesthetized with injection i.p. of ketamine and xylazine, following the method of Hamoy et al. (2018). For electrode implants, lidocaine was used as a local anesthetic (Hipolabor laboratory, Sabará, MG, Brazil). As a convulsant agent, PTZ was used; furthermore, an anticonvulsant agent, DZP, and an anticonvulsant inhibitor, FMZ, were used.

After the surgery process, the animals were allocated to individual cages. The electrodes were paired with a digital data acquisition system with a high impedance amplifier 5 days after surgery (Grass Technologies, P511), they were monitored by an oscilloscope (Protek, 6510), and data acquisition was performed by an acquisition board (National Instruments, Austin, TX, United States). Data were monitored and analyzed continuously at 1 kHz in a low pass of 3 kHz and a high pass of 0.3 Hz.

After drug application, 10 min of accommodation was expected, and the animals were carefully immobilized to avoid interference with the records. The recording of activity (EEG) lasted 15 min. This procedure was performed with all groups: control, EEOS (300 mg/kg i.p.), DZP (5 mg/kg i.p.) and PTZ (60 mg/kg i.p.). The DZP 5 mg/kg ip was given 10 min after receiving PTZ 60 mg/kg i.p. and EEOS 300 mg/kg i.p. Ten minutes after receiving 60 mg/kg PTZ i.p., recordings were started.

The possible mechanism of action was evaluated based on the application of a competitive antagonist of the BDZs, FMZ 0.1 mg/kg i.p., with subsequent recording lasting 15 min.

### Data Analysis

**FIGURE 1:**
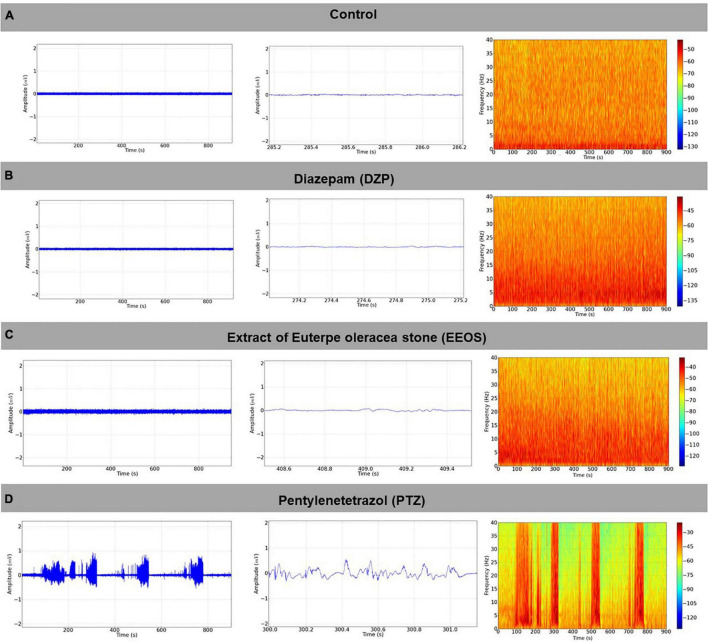
Baseline electroencephalogram (EEG) tracing, the signal obtained from the right hemisphere, using as a reference the left hemisphere with 1 s magnification (in the center), and the respective energy distribution spectrogram (right) **(A)**; electrocorticographic (ECoG) tracing after diazepam (DZP) administration (5 mg/kg i.p.) (left), with 1-s amplification (center) and power distribution spectrogram (right) **(B)**; ECoG record observed after the administration of extract of *Euterpe oleracea* stones (EEOS) (300 mg/kg i.p.), with magnification and spectrogram **(C)**, ECoG tracing after administration of pentylenetetrazol (PTZ) 60 mg/kg i.p., with amplification containing 1 s of the recording in the tracing, and a spectral energy distribution graph (spectrogram) **(D)** (900 s record length).

In the Python programming language (version 2.7), offline analysis was performed using a *de novo* tool. For mathematical processing, the “Numpy” and “Scipy” libraries were used, and for graphing and plotting, the “matplotlib” library was used. A graphical interface was selected using the PyQt4 library. The spectrograms were calculated using the 256-point Hamming window (256/1,000 s). For power spectral density (PSD), an overlap of 128 points per window was generated in each frame. For each chart, the Welch mean periodogram method was implemented to calculate the PSD. In addition, frequency histograms were obtained by calculating the PSD of the signal using the 256-point Hamming window without overlap, yielding a resolution of 1 Hz per box. The set of experiments is an average that corresponds to each wave displayed on the PSD. The individual boxes are representations of the calculated averages of the PSDs in each group (Figure 1D). Analyses were performed at frequencies up to 40 Hz and were divided into bands according to Jalilifar et al. (2017) in delta (1–4 Hz), theta (4–8 Hz), alpha (8–12), beta (12–28), and gamma (28–40 Hz) for interpretation of the dynamics during the development of seizures (Hamoy et al., 2018).

### Statistical Analysis

Since the residues were normally distributed and the variations were equal, comparisons between the mean amplitude of the tracings and the control values (Figures 1A–D) were made using one-way ANOVA and Tukey’s counter test. In all cases, the minimum significance level of *p* < 0.05 was analyzed by GraphPad ^®^ Prism 8.0 software.

## Results

### Phenolic Compounds Contents

The content of bioactive compounds from EEOS was as follows: total polyphenols 348.43 mg of gallic acid equivalents per gram of dried extract, flavanols 19.84 mg of catechin equivalents per gram of dried extract, flavonols 6.28 mg of myricetin-3-O-α-L-rhamnopyranoside equivalents, and condensed tannins 74.18 mg of cyanidin equivalents per gram of dried extract.

### Behavioral Stages

The behavioral characterization of EEOS anticonvulsant action (300 mg/kg i.p.) after PTZ administration (60 mg/kg i.p.) according to the order of the onset of behavior showed the progression of acute seizures to the third stage and they presented in order of appearance: immobility with latency 85.11 ± 6.528 s (Stage I), followed by rapid movements with vibrissae averaging 116.9 ± 12.25 s (Stage II) and isolated clonic seizures in the forelimbs with a mean latency of 170.0 ± 19.10 s (Stage III). There was no progression to stages IV and V, with the most severe seizures. The group that received pretreatment with DZP presented the characteristic behavior of stage II, with a latency of 282 ± 32.06 s (Table 1).

**TABLE 1:** Behavioral characterization of seizures triggered by the administration of pentylenetetrazol (PTZ) and after previous administration of diazepam (DZP) or extract of *Euterpe oleracea* stones (EEOS) (latency in seconds).

Stage	Behavior	PTZ 60 mg/kg i.p.	DZP 5 mg/kg + PTZ i.p.	EEOS 300 mg/kg + PTZ i.p.
0	No behavioral changes after PTZ administration.	–	–	–
I	The animal has immobility.	43.56 ± 6.560	–	85.11 ± 6.528
II	The animal exhibits rapid vibratory movements and a feathered tail.	58.00 ± 5.760	282 ± 32.06	116.9 ± 12.25
III	The animal presents isolated clonic seizures of one or more limbs, especially the anterior limbs.	89.33 ± 6.042	No evolution	170.0 ± 19.10
IV	The animal has generalized clonic seizures, abundant salivation, and transient loss of posture reflex.	103.2 ± 5.652	No evolution	No evolution
V	The animal presents tonic-clonic seizures (Great-mal), loss of posture reflex, and death from respiratory arrest.	165.0 ± 19.70	No evolution	No evolution

### The Basis for Electrocorticographic Analysis of Extracts of *Euterpe oleracea* Stone as an Anticonvulsant

**FIGURE 2:**
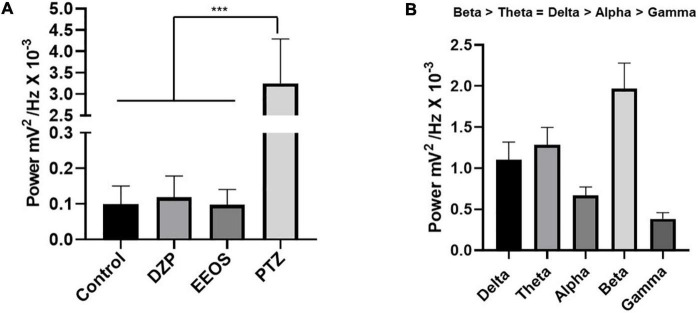
Average power in control electrocorticographic (ECoG) traces, DZP, extract of *Euterpe oleracea* stones (EEOS), and pentylenetetrazol (PTZ) **(A)**, and average power of brainwaves during convulsions caused by PTZ **(B)**. Different letters in columns show significant differences after ANOVA followed by the Tukey’s test (***p < 0.0001, *n* = 9).

After characterization of the convulsive stage, the efficiency of EEOS (300 mg/kg i.p.) was tested considering the deepening of the previous seizure tested stages. The EEG record traces of the control group, DZP, and EEOS presented higher powers in the low-frequency oscillations (0–10 Hz), as shown in the spectrograms (Figures 1A–C). When measuring the power of the obtained traces, the basal tracing with an average of 0.09926 ± 0.05109 mV^2^/Hz × 10^–3^ did not present a significant difference for the DZP and EEOS groups, which gave 0.1181 ± 0.06072 and 0.09754 ± 0.04330 mV^2^/Hz × 10^–3^, respectively. After PTZ administration, an increase in the characteristic amplitude could be observed in the electrocorticographic (ECoG) tracing of the seizures (Figure 1D). The average power significantly increased (*p* < 0.05) to 3.252 ± 1.041 mV^2^/Hz × 10^–3^ [*F*_(3, 32)_ = 81.71 *p* < 0.0001] (Figure 2A).

In the analysis of brainwaves (delta, theta, alpha, beta, and gamma), the predominant brain wave type during PTZ-induced seizures was beta, with an average of 1.967 ± 0.3135 mV^2^/Hz × 10^–3^, followed by theta (1.285 ± 0.2117 mV^2^/Hz × 10^–3^) and delta (1.100 ± 0.2205 mV^2^/Hz × 10^–3^), with no significant difference. Alpha (0.6697 ± 0.1014 mV^2^/Hz × 10^–3^) and gamma (0.3838 ± 0.07592 mV^2^/Hz × 10^–3^) were the least frequent (Figure 2B).

### Extracts of *Euterpe oleracea* Stone Anticonvulsant Potential

#### Açai Stones Have Anticonvulsant Potential and Are Effective in Reducing Pentylenetetrazol-Enhanced Preponderant Beta Waves

**FIGURE 3:**
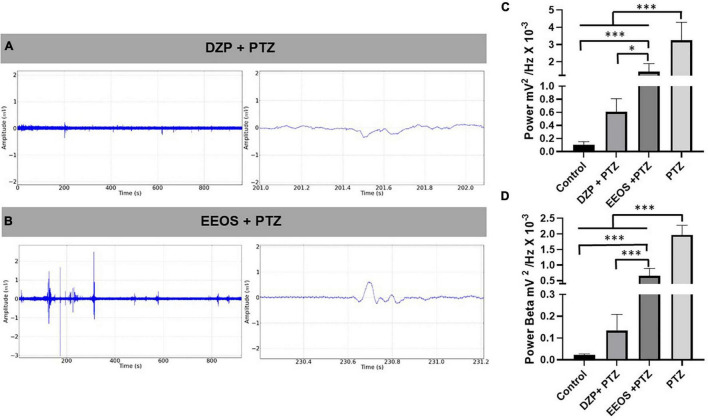
Electrocorticographic (ECoG) tracing of diazepam (DZP) **(A)** and extract of *Euterpe oleracea* stones (EEOS) **(B)**, seizure inhibition after pentylenetetrazol (PTZ) administration. **(C)** Total power averages of the control records, DZP + PTZ, EEOS + PTZ, and PTZ for PTZ-induced seizure outbreaks. **(D)** Demonstration of the potency of beta waves after administration of DZP + PTZ and EEOS + PTZ compared to the means obtained from the beta waves for PTZ. Different letters in columns show significant differences after ANOVA followed by Tukey’s test (*p < 0.01, ^***^p < 0.0001, *n* = 9).

To check the anticonvulsant action, PTZ (60 mg/kg) was injected 15 min after DZP (5 mg/kg i.p.) and EEOS (300 mg/kg) administration, and ECoG recordings at 15 min were used to observe the inhibition of seizure onset caused by PTZ. In this case, the recordings exhibited less amplitude discrepancy for DZP, but EEOS reduced the deflagrations compared to PTZ (Figures 3A,B). The linear power evaluation was 3.252 ± 1.041 mV^2^/Hz × 10^–3^ for PTZ, [*F*_(3, 32)_ = 51.16 *p* < 0.0001] and it was significantly reduced to 1.418 ± 0.4691 mV^2^/Hz × 10^–3^ after administration of EEOS and DZP group (0.6067 ± 0.1994 mV^2^/Hz × 10^–3^). The DZP group showed no statistical difference to the control group (0.09926 ± 0.05109 mV^2^/Hz × 10^–3^) (*p* = 0.155) (Figure 3C).

To assess beta force during PTZ-induced seizures, the baseline (0.02142 ± 0.006091 mV^2^/Hz × 10^–3^) and DZP + PTZ (0.1339 ± 0.07427 mV^2^/Hz × 10^–3^) showed no significant differences. The EEOS + PTZ group presented a higher value (0.6505 ± 0.2400 mV^2^/Hz × 10^–3^). The group that received only PTZ (1.967 ± 0.3135 mV^2^/Hz × 10^–3^) presented a significant difference for all groups [*F*_(3, 32)_ = 177.5; *p* < 0.0001] (Figure 3D).

### Flumazenil Reduces Seizure Control by Diazepam and Extracts of *Euterpe oleracea* Stone

**FIGURE 4:**
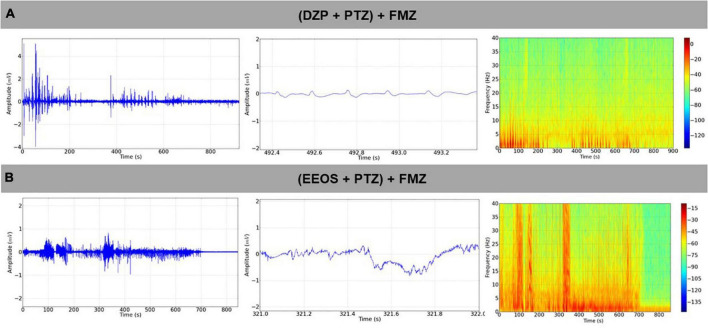
Electrocorticographic (ECoG) tracing after the antagonization of diazepam (DZP) action by flumazenil (FMZ) causing the reappearance of tracings compatible with a convulsive condition **(A)**, seizure reappearance after the administration of FMZ in extract of *Euterpe oleracea* stones (EEOS)-controlled seizures **(B)**.

**FIGURE 5:**
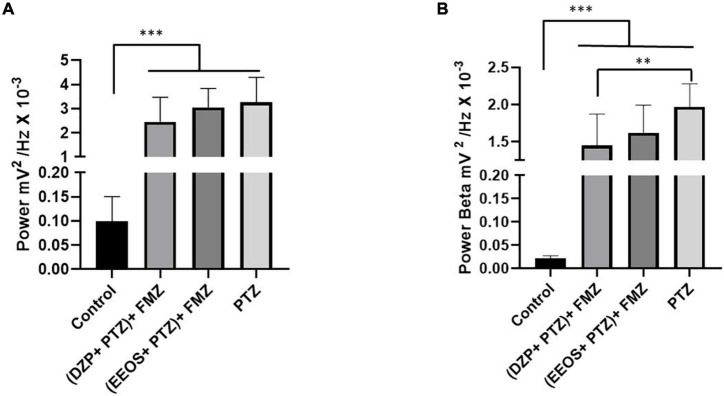
Power in the records after flumazenil (FMZ) use **(A)** and the power of beta waves after FMZ administration **(B)**. The significant differences are shown in different letters in the columns after ANOVA followed by Tukey’s test (^**^p < 0.001; ^***^p < 0.0001, *n* = 9).

Diazepam and EEOS showed efficacy in controlling seizures triggered by PTZ. Therefore, after controlling the seizures, FMZ, a BDZ antagonist of the GABAA receptor, was administered to elucidate the mechanism of action of EEOS. After administration of FMZ, a return to seizures could be observed for the DZP and EEOS groups. The ECoG tracings were compatible with seizures (Figures 4A,B), suggesting that the action of EEOS in the control of seizures involves the GABAA receptor. These data can be analyzed from the power observed on the records; for the basal control, the mean power was 0.09926 ± 0.05109 mV^2^/Hz × 10^–3^, which maintained a lower value [*F*_(3, 32)_ = 27.81; *p* < 0.0001] than the other groups. The (DZP + PTZ) + FMZ group presented an average of 2.453 ± 1.018 mV^2^/Hz × 10^–3^, (EEOS + PTZ) + FMZ 3.052 ± 0.7742 mV^2^/Hz × 10^–3^ and PTZ 3.252 ± 1.041 mV^2^/Hz × 10^–3^. No significant difference was seen between DPZ and EEOS in the power records (Figure 5A) or beta waves (Figure 5B).

Regarding beta waves, the basal control presented a value of 0.02142 ± 0.006091 mV^2^/Hz × 10^–3^ showed statistical differences for all groups [*F*_(3, 32)_ = 63.73; *p* < 0.0001]. The (DZP + PTZ) + FMZ group (1.453 ± 0.4200 mV^2^/Hz × 10^–3^) showed no significant difference from the (EEOS + PTZ) + FMZ group (1.618 ± 0.3732 mV^2^/Hz × 10^–3^). The PTZ group exhibited a higher value for beta waves (1.967 ± 0.3135 mV^2^/Hz × 10^–3^). The PTZ group exhibited a higher value for beta waves (1.967 ± 0.3135 mV^2^/Hz × 10^–3^) and presented a statistical difference for the (DZP + PTZ) + FMZ group (*p* < 0.001).

## Discussion

Behavioral assessment and electrophysiological recordings are helpful to compare changes caused by neuronal excitability that trigger seizures in chemoconvulsant models (Ono et al., 1990; Hamoy et al., 2018). Our EEOS data showed that in the behavioral assessment, the increase in latency for seizures and the non-appearance of behavioral changes, such as widespread clonic seizures with transient loss of the posture reflex and tonic-clonic seizures with loss of postural reflex after PTZ administration, indicate that EEOS compounds are effective in increasing the seizure threshold for the PTZ model, which was confirmed by the ECoG recording, considering that behavioral changes could not be observed. These recordings showed a decrease in peak and wave energy.

The GABAA receptor causes decreased excitability of neurons by increasing the conductance of chloride ions due to increasing the chloride channel opening frequency and subsequent neuronal membrane hyperpolarization (Aburawi, 2021).

According to our previous study (Muto et al., 2021), EEOS exhibited strong sedative and muscle relaxant effects in animal models, as demonstrated by the behavioral characterization and electromyographic records. However, the potential use of EEOS as an anticonvulsant and its pharmacodynamic route are still poorly understood. According to Souza-Monteiro et al. (2015), the previous use of clarified açai juice was able to reduce the intensity of the convulsions caused by PTZ. Souza-Monteiro et al. (2019) also evaluated the antidepressant activity of clarified açai. The experiments carried out with the fruit of *Euterpe oleracea* were important in the evaluation of consumed composites; however, other parts of the fruits, such as stones, were still ignored. To better understand the underlying mechanisms that trigger the anticonvulsant activity of EEOS, different modulatory drugs (DZP, PTZ, FMZ) at GABAA receptors were comparatively evaluated by EEG.

The CNS depressant effect of EEOS was previously observed by the reduction in both motor/cardiac activity, myorelaxation, and respiratory depression due to the presence of bioactive constituents such as tannins, flavonoids, procyanidin, catechin, polymeric proanthocyanidins, cinnamtannin, and other phenolic compounds (Muto et al., 2021). Tannins have a similar depressant effect in the CNS, and due to their dose-dependent action, which reduces locomotor activity, tannins have a role in potentiating phenobarbital-induced sleeping time. In another study were also reported to have anxiolytic and anticonvulsant effects on mice of flavonoids, linalool, and α-tocopherol presents the extract of leaves of *Cissus sicyoides* L. (Vitaceae) (Almeida et al., 2009).

Similar results were obtained after administration of the *Curcuma longa* essential oils at 100 mg/kg i.p. in which curcumol resulted in anticonvulsant activity, controlling PTZ-induced seizures by reducing seizure severity (Ding et al., 2014). Bioactive compounds promote activation of the GABAA receptor at a site independent of BDZs in hippocampal neurons, suppressing neuronal networks (Liu et al., 2017).

Other essential oil derivatives of plants that have pharmacological action in seizure control through modulation of GABAergic neurotransmissions, such as *Dennettia tripetala* oil due to its constituent, 1-nitro-2-phenylethane, interact with BDZ receptors (Oyemitan et al., 2013; Abbasi et al., 2017). This mechanism is similar to that observed for EEOS activity in the gamma subunit of the chlorine ionophore, allosterically potentiating GABA. However, additional studies are necessary to correlate the EEOS with GABAergic targets.

Troponin-4-ol and thymol are monoterpenes that have seizure control mechanisms mediated through bioactive compounds from plants, such as eugenol, and they might increase the seizure threshold through the modulation of ion channels (Nóbrega et al., 2014; Sancheti et al., 2014).

Another bioactive compound, α-asarone, reduces neuronal excitability by increasing GABAergic activity in animal models (Huang et al., 2012). These compounds can act as protectors by reducing the inflammatory process induced by seizures, as observed in α-asarone, borneol, carvacryl acetate, eugenol, verbenone, β-caryophyllene, and nerolidol (Pires et al., 2015; Kaur et al., 2016; Tambe et al., 2016; de Melo et al., 2017; Joushi and Elahdadi Salmani, 2017). Thus, EEOS controlled PTZ-induced seizures, but its myorelaxant action should not be disregarded (Muto et al., 2021), demonstrating a similar spectrum of action to that of BDZs.

The allosteric enhancement of the inhibitory actions of GABA is associated with the binding of BDZs to the modulatory site located between the α1, α2, α3 or α5 and γ subunits. The α subunit subtype of the GABAA receptor is associated with the BDZ clinical effect. The α1 subunit mediates the sedative and amnestic actions of BDZs, the α2 and α3 subunits, the anxiolytic action of BDZs (D’Hulst et al., 2009) and the α5 subunit is related to memory/learning impairment activity (Collinson et al., 2002).

As BDZs lack intrinsic activity at the GABAA receptor in the absence of GABA, the old terminology used to describe agonists (e.g., DZP), antagonists (e.g., FMZ), and inverse agonists (e.g., FG 7142) are sometimes replaced by the terms positive allosteric modulators, neutral allosteric modulators, and negative allosteric modulators, respectively (Hood et al., 2014).

**FIGURE 6:**
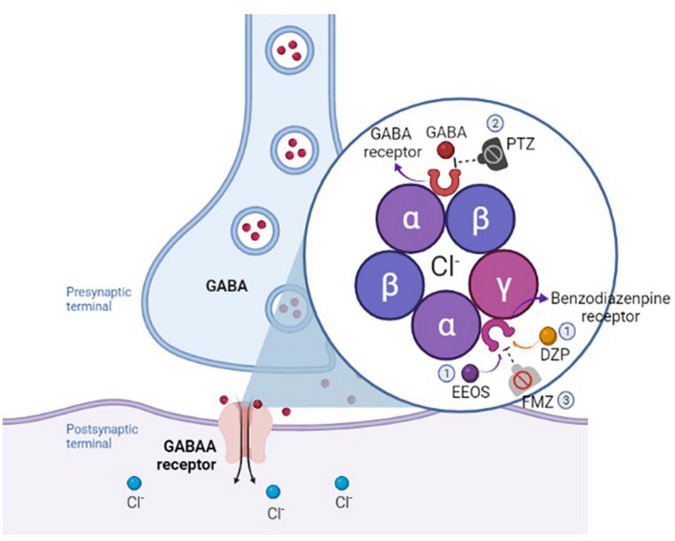
Schematic illustration of the mechanism of extract of *Euterpe oleracea* stones (EEOS) or diazepam (DZP) on benzodiazepine (BDZ) receptor (1), followed by pentylenetetrazol (PTZ) antagonism on GABAA receptor (2), and flumazenil (FMZ), a competitive antagonist of the effect of benzodiazepine ligand (3).

Pentylenetetrazol is a GABAA receptor antagonist, a selective chloride channel blocker (Mahomed and Ojewole, 2006; Szyndler et al., 2006). To investigate the mechanism by which EEOS is responsible for reducing seizures, FMZ, a competitive antagonist of the effect of BDZ drugs on the CNS, was used to observe the electrophysiological changes in the ECoG and analyze its potency. After the administration of FMZ, all of the EEOS and DZP groups showed a resumption of the seizures. Hence, it is possible that activation of the BDZ site or another receptor modulated by EEOS decreases the intensity of PTZ-induced seizures (Figure 6).

Flumazenil acts as a neutral GABAA modulator at low doses and in the presence of BDZs such as DZP. First, although FMZ influences all GABAA receptor subtypes as an antagonist, it has particular partial positive allosteric modulatory activity at the α6 subunit of GABAergic receptors (Hood et al., 2014). Its antagonism to the sedative properties of a wide range of BDZs, such as midazolam, DZP, and lorazepam, occurs when it competitively displaces BDZ molecules by binding to the extracellular surface of GABAA receptors, preventing BDZ from binding to the same site (Sivilotti, 2016).

The mediation of muscle relaxation and sedation is *via* the α1 receptor, the anxiolysis and anticonvulsant effects, α2 or α3, and amnesia at the extrasynaptic receptor with α5. Therefore, FMZ has a neutral allosteric modulating action at the BDZ site, with selective antagonism at α1 and partial agonism at α2, α3, and α5 (Sivilotti, 2016). The EEOS ligand receptor, according to the behavioral and electrophysiology records, specifically targets the GABAA α1 receptor, which mediates sedation and muscle relaxation, and anxiolysis and anticonvulsant effects *via* α2 or α3.

The suggested mechanism of EEOS is closely related to agonist activity against the GABAA receptor complex and hence may act like BDZ-like molecules. The EEOS ligand for GABAA receptors promotes increased chloride ion flux conductance, inhibiting the firing of new action potentials and resulting in the induction of sedative and anticonvulsive properties through this channel (Figure 6). The inhibition that reduces changes in membrane excitability creates a neuroprotective action against depolarization caused by PTZ or other stimuli.

## Conclusion

In the present study, EEOS reduced the intensity of PTZ-induced seizures outbreaks, possibly exerting its ligand action on the GABAA receptor *via* BDZ subunits. Its anticonvulsant activity was inhibited by FMZ, a BDZ receptor antagonist, indicating competition for the same locus of the BDZ GABAA receptor.

## Data Availability Statement

The raw data supporting the conclusions of this article will be made available by the authors, without undue reservation.

## Ethics Statement

The animal study was reviewed and approved by the Committee for Ethics in Experimental Research with Animals of Federal University do Pará (Brazil; license no: 1089220518).

## Author Contributions

NM: conceptualization and writing. MH: methodology and software. CS: investigation. AH: visualization. DL: original draft preparation. VM: validation. HR: reviewing and editing. All authors read and approved the final manuscript.

## Conflict of Interest

The authors declare that the research was conducted in the absence of any commercial or financial relationships that could be construed as a potential conflict of interest.

## Publisher’s Note

All claims expressed in this article are solely those of the authors and do not necessarily represent those of their affiliated organizations, or those of the publisher, the editors and the reviewers. Any product that may be evaluated in this article, or claim that may be made by its manufacturer, is not guaranteed or endorsed by the publisher.

## Footnotes

1
http://oacu.od.nih.gov/regs/index.htm
